# An Improved Method for Accurate Radiation Measurement Based on Dark Output Noise Drift Compensation

**DOI:** 10.3390/s23136157

**Published:** 2023-07-05

**Authors:** Baolin Zhao, Kaihua Zhang, Yaxin Yu, Kun Yu, Yufang Liu

**Affiliations:** Henan Key Laboratory of Infrared Materials & Spectrum Measures and Applications, School of Physics, Henan Normal University, Xinxiang 453007, Chinayukun@htu.edu.cn (K.Y.)

**Keywords:** fiber optic spectrometer, dark output noise, signal drift, drift correction

## Abstract

This paper verified through experiments that change in ambient temperature are the main cause of dark output noise drift. Additionally, the impact of dark output noise drift in fiber optic spectrometers on emissivity measurements has been investigated in this work. Based on an improved fiber optic spectrometer, two methods were proposed for characterizing and correcting the dark output noise offset in fiber optic spectrometers: the mean correction scheme and the linear fitting correction scheme. Compared to the mean correction scheme, the linear fitting correction scheme is more effective in solving the problem of dark output noise drift. When the wavelength is greater than 1600 nm, the calibration relative error of silicon carbide (SIC) emissivity is less than 0.8% by the mean correction scheme, while the calibration relative error of silicon carbide emissivity is less than 0.62% by the linear fitting correction scheme. This work solves the problem of dark output noise drift in prolonged measurement based on fiber optic spectrometers, improving the accuracy and reliability of emissivity and quantitative radiation measurement.

## 1. Introduction

The fiber optic spectrometer is a versatile spectral analysis tool that is widely used for its ability to study the optical characteristics of a target. This tool enables the recording of absorption spectra, reflection spectra, or emission spectra of materials, which provides valuable information about the properties of a material. Emissivity is a fundamental material property that describes the efficiency with which a material emits radiation. It is an important parameter in many applications where radiation exchange occurs, such as heat transfer, radiation temperature measurement, and thermal imaging. Due to the advantages such as flexible, portable, and real-time monitoring of the fiber spectrum, a large number of scientific researchers have used the fiber optic spectrometer to study the material radiation characteristics [1,2,3,4,5,6,7,8,9,10,11].

In the processes of scientific production, the spectral information of the product can be obtained through fiber optic spectrometers, and the quality of the product can be detected quickly [12,13]. Some literature has reported temperature measurements by using fluorescence spectroscopy in specific scenarios [14,15,16,17]. In addition, it has been reported that using fiber optic spectrometers to study the polarization spectrum of an oil slick on the sea and monitoring the pollution of an oil slick on the sea surface through polarization spectrum [18,19]. The fiber optic spectrometers, as radiation signal measurement equipment, also have a wide range of applications in measuring emissivity and temperature on object surfaces [7,8,9,10,20,21,22,23,24]. Qiang Lu et al. reported the temperature and emissivity of flame during biomass combustion, which is studied by utilizing a fiber optic spectrometer [8]. Weijie Yan et al. studied related work of the emissivity and the temperature of fuels containing alkali metals during combustion [7,24]. The increasing demand for temperature measurement scenarios has prompted researchers to engage in radiation temperature measurement research, and multi-wavelength temperature measurement methods and devices that use fiber optic spectrometers as spectral signal capture devices have been reported [25,26]. Some devices used for in situ radiation measurement have also been applied in complex industrial environments [6,7,8,9,24,25,26,27,28].

It is well-known that the photodetectors are particularly susceptible to temperature fluctuations. The random noise of thermal noise and dark current noise increases with the increase in temperature, which will cause the detector to output higher levels of noise and reduce its sensitivity. The complex industrial environments may mean that the surrounding environment is variable; changes in the ambient temperature will affect the inner temperature of the fiber optic spectrometer, which will cause the detector to generate different degrees of signal drift. A Hijazi et al. proposed a calibrated dual-wavelength infrared thermometry approach with non-greybody for metal machining temperature measurements [26]. During the experiment, the author found that the background reading, which is also called dark output noise in other places, was fairly sensitive to the temperature of the objective lens body. To compensate for this, background readings were taken at uniform time intervals during the experiments. Wang Hangzhou et al. developed a fiber optic–based spectrometry system to achieve automatic long-term measurements of spectral irradiance in sea ice environments [29,30,31]. The author uses linear fitting to establish a corresponding model for dark output noise. The temperature effect on spectrometer dark output within the visible wavelength range has been reported in detail.

The emissivity measurement error and the multi-wavelength temperature measurement error, which are caused by the deviation of the measurement radiation signal, are noteworthy issues when the fiber optic spectrometer is used for quantitative measurement. Wang Nian et al. pointed out that the inversion temperature deviation of multispectral thermometry increases with the increase of emissivity deviation [32]. The radiation of the measured object is one of the direct data in a temperature inversion, and according to the Planck formula, its deviation directly affects the temperature inversion results. In algorithm research of multispectral thermometry, most researchers add a maximum of 5% random error to test the algorithm’s anti-interference ability [33,34]. Large radiation deviations will reduce the accuracy of temperature inversion. For the research conducted in the laboratory, the impact of dark output noise drift can be ignored due to the controllable environmental conditions. Nevertheless, only a few studies have mentioned the impact of temperature on the dark output noise drift in prolonged measurements carried out in complex environments. Therefore, it is meaningful to study the influence of temperature on dark output noise and the influence of dark output noise drift on emissivity measurement.

This paper investigates the impact of dark output noise drift on emissivity measurement. In order to solve the problem of dark output noise drift in the infrared detector of the fiber optic spectrometer, an improved scheme for the fiber spectrometer is proposed. The problem of dark output noise drift is addressed through the implementation of the mean model and the linear fitting model in this study. The comparison of these two models in their ability to correct dark output noise drift is presented. The paper uses the object emissivity relative error to replace the object radiation relative error. This method can not only verify the accuracy of measuring radiation by the device but also convert emissivity relative error to radiation relative error equivalently. The findings reveal that both correction schemes are effective in addressing the impact of dark output noise drift when the drift is smaller. While as the drift increases, the linear fitting correction model outperforms the mean model. The calibration relative error of silicon carbide (SIC) emissivity is below 1% with both correction schemes at 1600–2500 nm, among which the mean correction scheme resulted in a calibration relative error of emissivity lower than 0.8%, while the linear fitting correction scheme resulted in a calibration relative error of emissivity lower than 0.62%.

## 2. Correction Principle and Correction Device

### 2.1. The Principle of Dark Output Noise Drift Correction

In the actual measurement, the output signals of a fiber optic spectrometer not only contain signals that are responded to by the radiations emitted from the sample but also contain the signal of dark output noise. Thus, the total signal *S_all_* obtained by the fiber optic spectrometer at temperature *T* is given by

(1) $$S_{all}=V_{d}\left(\Delta t,T_{amb}\right)+S\left(\lambda ,T\right)\Delta t$$

where *V_d_*(Δ*t*,*T_amb_*) is the dark output noise signal of the detector at *T_amb_*, which is related to the integration time Δ*t* and ambient temperature *T_amb_*, and increases with the increase of the integration time. *S*(*λ*,*T*) is the radiation signal emitted from the object. The dark output noise signal can be obtained when there is no signal input. When the ambient temperature changes, the dark output noise of the fiber optic spectrometer will drift. While using a spectrometer again to measure the same temperature object, the total radiation signal ${S^{\prime }}_{all}$ can be expressed as

(2) $${S^{\prime }}_{all}=V_{d}\left(\Delta t,T_{amb}\right)+S^{\prime }\left(\lambda ,T\right)\Delta t$$

where $S^{\prime }\left(\lambda ,T\right)$ is the radiation signal containing dark output noise drift, which can be expressed as $S^{\prime }\left(\lambda ,T\right)=S\left(\lambda ,T\right)+\Delta V_{d}/\Delta t$. Δ*V_d_* is the signal of the dark output noise drift. The dark output noise signal is typically obtained once prior to measurement when utilizing a fiber optic spectrometer. In situations where the fiber optic spectrometer is utilized for a brief period, or the ambient temperature remains relatively constant, the dark output noise drift can be ignored. However, when the fiber optic spectrometer is employed continuously as a signal capture device, the drift of its dark output noise cannot be ignored. In Formula (1), when the measured target is a standard blackbody radiation source, and the background radiation is not considered, then the following relationship exists:

(3) $$S_{bb}\left(\lambda ,T\right)=\frac{S_{all-bb}-V_{d}\left(\Delta t,T_{amb}\right)}{\Delta t}=R\left(\lambda \right)L_{bb}\left(\lambda ,T\right)$$

where *S_bb_*(*λ*,*T*) is the radiation signal entering the detector, *S_all-bb_* is the total signals of fiber optic spectrometer when measuring blackbody. *R*(*λ*) is the response function of the measurement apparatus, which is expected as a function of the wavelength and independent of temperature. It is affected by the photoelectric conversion coefficient of the spectrometer, the reflection coefficient of the mirrors in the optical path, and the transmission coefficient of the incidence window. *L_bb_*(*λ*,*T*) is the radiation of the blackbody at temperature *T*, and it can be calculated by the Planck formula. By measuring the standard blackbody radiation source, the response function of the measuring apparatus can be obtained. When measuring the radiation signal of the sample while considering the dark output noise drift, the radiation of the sample can be expressed as

(4) $$S_{s}\left(\lambda ,T\right)={S^{\prime }}_{s}\left(\lambda ,T\right)-\Delta V_{d}/\Delta t$$

where ${S^{\prime }}_{s}\left(\lambda ,T\right)$ is the sample radiation signal containing dark output noise drift, *S_s_*(*λ*,*T*) is only the sample radiation signal that enters the detector. The emissivity of sample ε(*λ*,*T*) can be given by the Formula (5) according to the emissivity definition:

(5) $$\varepsilon \left(\lambda ,T\right)=\frac{L_{s}\left(\lambda ,T\right)}{L_{bb}\left(\lambda ,T\right)}=\frac{\frac{S_{s}\left(\lambda ,T\right)}{R\left(\lambda \right)}}{\frac{S_{bb}\left(\lambda ,T\right)}{R\left(\lambda \right)}}=\frac{S_{s}\left(\lambda ,T\right)}{S_{bb}\left(\lambda ,T\right)}=\frac{{S^{\prime }}_{s}\left(\lambda ,T\right)-\Delta V_{d}/\Delta t}{S_{bb}\left(\lambda ,T\right)}$$

where *L_s_*(*λ*,*T*) is the radiation of sample radiation. It can be seen from Formula (5) that if the dark output noise drift is ignored during radiation measurement, the real emissivity of the sample will be affected. Combined with Planck’s formula, it will cause different degrees of deviation to the temperature inversion when the emissivity changes. In addition, the emissivity of the sample can also be obtained by theoretical calculation based on the Fresnel formula:

(6) $$\varepsilon =\frac{4n}{{\left(n+1\right)}^{2}}$$

where *n = n*_1_/*n*_2_, *n*_1_ and *n*_2_ are the refractive indices of air and sample, respectively. The relative error of emissivity *δ* can be calculated by Formula (7):

(7) $$\delta =|\frac{{S^{\prime }}_{s}\left(\lambda ,T\right)}{S_{bb}\left(\lambda ,T\right)}-\frac{S_{s}\left(\lambda ,T\right)}{S_{bb}\left(\lambda ,T\right)}|/\frac{S_{s}\left(\lambda ,T\right)}{S_{bb}\left(\lambda ,T\right)}\times 100\%=\frac{|\Delta V_{d}/\Delta t|}{S_{s}\left(\lambda ,T\right)}\times 100\%$$

It can be seen from Formula (7) that the relative error of emissivity is numerically equivalent to the relative error of the sample radiation signal. Therefore, we can understand its impact on the relative error of the sample radiated signal by exploring the effect of dark output noise drift on the relative error of the sample emissivity.

### 2.2. Correction Device

**Figure 1:**
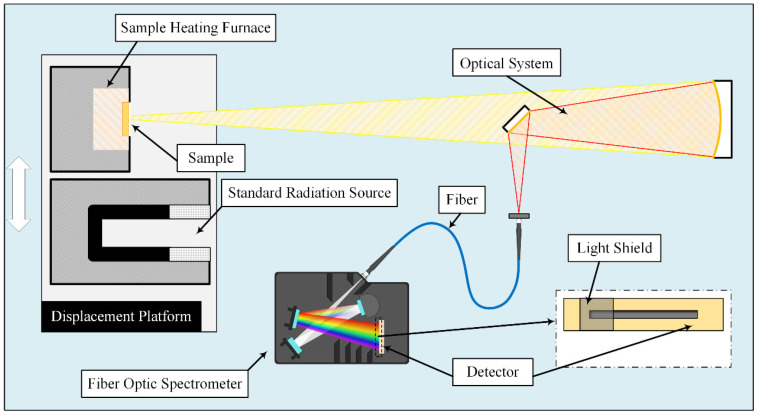
The diagram of the emissivity measurement apparatus.

The emissivity measurement system was built based on the measurement principle of the energy contrast method, as shown in Figure 1. And it is mainly composed of a sample heating furnace, a standard radiation source, an optical system, and an improved fiber optic spectrometer [35]. The radiation coming from samples or blackbody is converged by a concave mirror, then reflected by a plane mirror, and finally coupled into the optical fiber, and enters the fiber optic spectrometer through a fiber with a diameter of 400 nm. All mirrors in the optical system are gold-coated mirrors; these mirrors offer > 96% average reflection in the infrared spectral range from 0.8–20 µm. The fiber optic spectrometer uses high-speed InGaAs linear image sensors with two-stage TE-cooled, covering the 0.9–2.5 µm wavelength range. There is a light shield above the linear image sensors to block the spectral signals in the range of 0.9–1.2 µm.

The medium-temperature blackbody (ISOTECH R970) with an effective emissivity higher than 0.995 is selected as the standard radiation source. The heating furnace employs a SIC heating element that is enclosed within a cylindrical chamber and insulated with glass fiber thermal insulation material. The furnace is designed to heat samples with a diameter of up to 50 mm. A high-precision PID controller is employed to heat the furnace inside to a maximum temperature of 1300 °C, while a type S thermocouple is used to measure the temperature fluctuations. To ensure optimal contact between the sample and the heating body, symmetrical clamps are positioned to secure the sample, with the heating body surfaces being finely polished with sandpaper. Heat transfer is the primary mechanism used to heat the sample during the experiment. The surface temperature of the sample was monitored by a high-precision colorimetric pyrometer, with its maximum temperature being approximately 850 °C.

## 3. Exploration of Dark Output Noise Drift

When using the fiber optic spectrometers, the phenomenon of dark output noise drift was found. In order to verify whether this phenomenon is related to the change in ambient temperature, a set of experimental schemes was designed. First, the domestically developed fiber optic spectrometer was placed in a chamber and blocked the incident slit of the fiber optic spectrometer to ensure that no external radiation entered the fiber optic spectrometer. Then, changing the temperature of the chamber and the dark output noise of the fiber optic spectrometer at different integration times was recorded every 2 min. At the beginning of the experiment, the internal temperature of the chamber was 15 °C, which was gradually increased to 35 °C and maintained for a period of time before rapidly increasing to 42 °C. Subsequently, the temperature of the chamber naturally cooled to room temperature.

**Figure 2:**
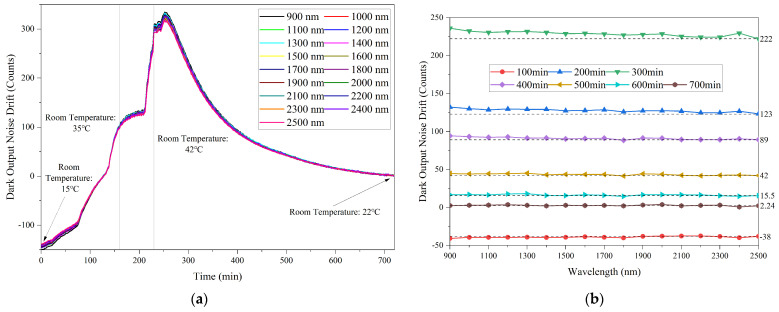
The dark output noise drift. (**a**) The dark output noise drift at different wavelengths as a function of time (Integration time:15 ms); (**b**) The dark output noise drift at different times as a function of wavelength (Integration time: 15 ms).

In order to obtain the drift of the dark output noise of the fiber optic spectrometer, based on the dark output noise at room temperature, the deviation is calculated. All the dark output noise data with different integration times are displayed as normalized data. Figure 2a shows the dark output noise drift at different wavelengths with an integration time of 15 ms as a function of time. It can be seen that as the ambient temperature increases, the dark output noise drift at different wavelengths gradually increases. Conversely, as the ambient temperature decreases, the dark output noise drift decreases gradually. The trend of dark output noise deviation is the same at different wavelengths. Although there are some differences in the dark output noise at different wavelengths at the same moment, this difference becomes more obvious as the temperature difference from room temperature increases.

This phenomenon can be observed more clearly in Figure 2b. Figure 2b shows the dark output noise drift as a function of wavelength at different times with an integration time of 15 ms. It can be seen that the dark output noise drift decreases as the wavelength increases when the drift is greater than 0, while the dark output noise drift increases as the wavelength increases when the drift is less than 0. It is worth emphasizing that the tendency becomes more obvious as the temperature difference from room temperature increases.

**Figure 3:**
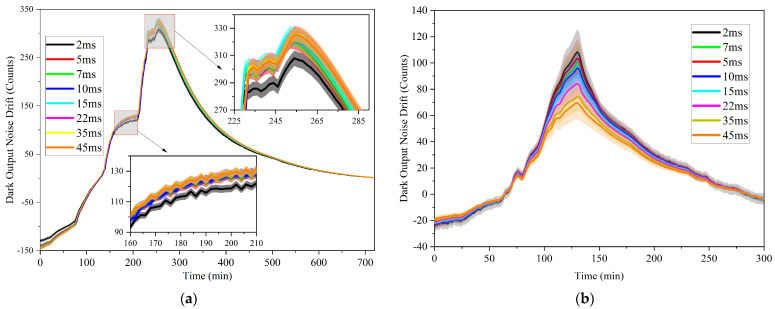
The dark output noise drift at different integration time as a function of time. Where (**a**) is the data on domestic equipment, (**b**) is the data on imported equipment.

To fully evaluate the drift of the dark output noise, both the mean and the standard deviation of the dark output noise drift data were calculated at different wavelengths. The results are presented in Figure 3a. Using the same test scheme to study the dark output noise drift of the imported fiber optic spectrometer, the mean and the standard deviation of the dark output noise drift data at different wavelengths measured by the imported fiber optic spectrometer are shown in Figure 3b. It is observed that the dark output noise drift trend of both the domestic and imported fiber optic spectrometer is similar under different integration times, albeit with varying numerical differences. Furthermore, it is noted that the standard deviation of dark output noise drift derived from both domestic and imported devices progressively increases with the rise in temperature difference.

The study on the dark output noise drift of fiber optic spectrometers shows that the change in ambient temperature will cause the dark output noise drift of fiber optic spectrometers, whether domestic or imported. The change of the room temperature during prolonged use in industrial production can greatly impact the accurate measurement of radiation signals. However, accurately establishing the functional relationship between room temperature and dark noise output can be challenging due to the lag in internal temperature changes in the fiber optic spectrometer. As shown in Figure 3, the dark output noise drift at different wavelengths is relatively consistent under the same integration time. Based on this consistency, a novel approach is proposed to enhance the accuracy of radiation signal capture in domestic fiber optic spectrometers. This approach obtains the dark noise drift data by blocking the detector signal in the wavelength range of 900–1200 nm. It then utilizes the average value of those dark noise drift data to correct for deviations in the signal caused by changes in environmental temperature. This correction eliminates signal deviations caused by changes in ambient temperature and thereby improves measurement accuracy.

## 4. The Influence of Dark Output Noise Drift on Emissivity

To investigate the influence of the dark output noise drift on radiation measurements, the emissivity of silicon carbide was measured at a constant temperature in the laboratory; multiple measurements were taken by altering the ambient temperature of the fiber optic spectrometer, and the resultant deviation in emissivity was calculated to validate the influence of dark output noise drift on the radiation signal. The reason for choosing silicon carbide as the test sample is that its emissivity is relatively stable and almost unaffected by temperature. In addition, the optical parameters and references of silicon carbide are relatively rich, which is helpful for comparing experimental results. The silicon carbide sample has a diameter of 50 mm and a thickness of 2 mm. Before the experiment, the surface of the sample was cleaned with absolute ethanol to ensure that the surface was free of impurities.

In the experiment, the radiation signal of silicon carbide with a surface temperature of 550 °C and the blackbody radiation signal at the same temperature was measured at a room temperature of 20 °C, and the dark output noise signal at the current state was recorded. The dark output noise signal recorded at 20 °C was utilized as the background noise of the fiber optic spectrometer. Changing the ambient temperature of the fiber optic spectrometer, the radiation signal of silicon carbide and the dark output drift signal at the same time were recorded at the ambient temperature of 15 °C, 25 °C, 0 °C, and 35 °C, respectively. Throughout the measurement process, the surface temperature of silicon carbide was maintained at 550 °C.

**Figure 4:**
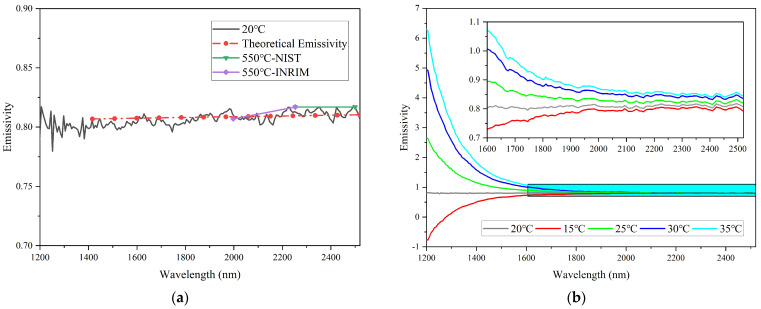
(**a**) The comparison of measured spectral emissivity at 550 °C when the ambient temperature of spectrometer is 20 °C, theoretical emissivity at 550 °C, and the emissivity at same temperature reported by other researchers. (**b**) The uncorrected spectral emissivity of silicon carbide as a function of wavelength at different ambient temperatures.

The spectral emissivity of silicon carbide at 550 °C calculated by Formula (5), where the ambient temperature of the spectrometer is 20 °C, and the theoretical emissivity of silicon carbide calculated by Formula (6) has been shown in Figure 4a. Figure 4a also shows the emissivity reported by B. Hay et al. [36]. As can be seen, the emissivity of silicon carbide is well consistent with the data reported by the National Institute of Standards and Technology (NIST) and the National Metrology Research Institute (INRiM). The same conclusion is also agreed with the relation between the theoretical emissivity and the measured emissivity without dark output noise drift. These data have well verified the reliability of our apparatus.

The uncorrected emissivity of silicon carbide can be obtained when ignoring the dark output noise drift Δ*V_d_* according to Formula (5) and shown in Figure 4b. When the ambient temperature changed, it can be seen that an increase in ambient temperature resulted in a gradual increase in the emissivity of silicon carbide, while a decrease in ambient temperature led to a decrease in its emissivity. In addition, the larger the changes in ambient temperature, the more significant the emissivity deviation. Notably, the uncorrected emissivity of silicon carbide increased as the wavelength decreased, and the value near the short wave exceeded 1, which violates the definition of emissivity. Planck’s formula states that the peak wavelength of the radiation signal shifts towards longer wavelengths as the temperature decreases. When the temperature of silicon carbide is low, the emitted radiation near the short wave is small, and even a small deviation in the signal can result in significant measurement errors. This phenomenon explains why the emissivity is greater than 1. The accuracy and repeatability of industrial online continuous measurement systems were seriously affected by the dark output noise drift of fiber optic spectrometers.

## 5. Dark Output Noise Drift Correction

**Figure 5:**
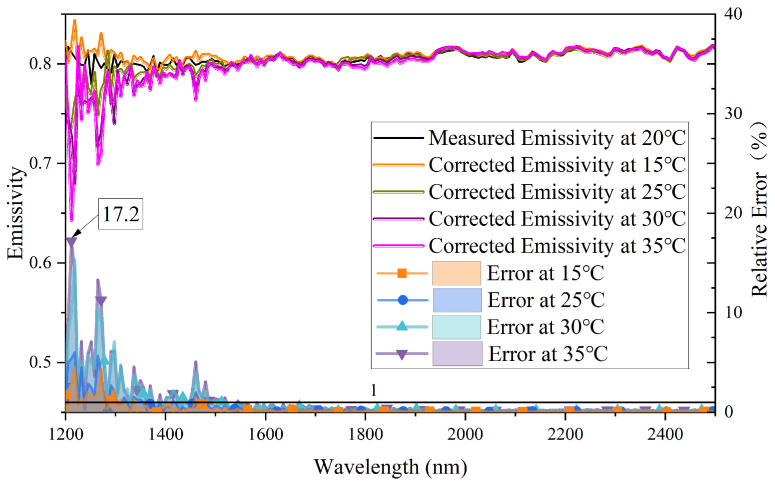
The spectral emissivity of silicon carbide corrected by mean correction scheme at different ambient temperatures and relative error of it.

In the acquired radiation signal, there are some diffracted lights entering the detector before 1200 nm; therefore, the data in the wavelength range of 900 to 1100 nm is chosen to correct the drift of dark output noise. From the prior research on dark output noise drift, the spectral signal can be corrected by utilizing the mean value of dark output noise. Firstly, the mean value of dark output noise in the wavelength range of 900–1100 nm is determined, and then the mean value is subtracted from the signal obtained by a fiber optic spectrometer in the wavelength range of 1200–2500 nm. The emissivity of silicon carbide is calculated again using the corrected spectral data. The corrected silicon carbide emissivity and the relative error of corrected emissivity are illustrated in the upper and lower parts of Figure 5, respectively. Table 1 shows the relative error of corrected emissivity by mean correction scheme at different wavelength ranges. As can be seen from Figure 5, the corrected emissivity measured at different ambient temperatures is almost the same, and the relative error of the corrected emissivity is within 1% when the wavelength is over 1600 nm. However, the relative error of corrected emissivity gradually increases as the wavelength decreases. In the wavelength range of 1200–1450 nm, the maximum relative error of corrected emissivity is 17.2%. Table 1 shows that the maximum relative error of corrected emissivity in the wavelength range of 1450–1600 nm is 5.06%, while when the wavelength is greater than 1600 nm, the relative error of the corrected emissivity is lower than 0.8%.

According to Planck’s formula, when the object’s temperature is low, the radiation emitted at short waves is almost zero. Hence, small deviations can cause significant relative errors. Furthermore, as shown in Figure 5, as the temperature difference in the measurement environment increases, the relative error of emissivity also increases obviously. Overall, the mean correction scheme can be employed to enhance the accuracy and repeatability effectively of the fiber optic spectrometer in the long wavelength range.

In actuality, it was observed that the dark output noise drift exhibited a distinct wavelength dependence, as demonstrated in Figure 2b. The drift decreased as the wavelength increased when the deviation was greater than 0, while the drift increased with wavelength when the deviation was less than 0. This indicates that the approach of employing the mean value to correct the dark output noise drift is inadequate. To address this issue, the method of linear fitting was utilized to establish the relationship between the dark output noise drift and wavelength. In the experiment of dark output noise drift, 360 sets of dark noise drift data were recorded at each integration time, resulting in a total of 2880 sets of dark noise drift data across eight different integration times. By fitting these data in the wavelength range of 1200–2500 nm with a linear model (*f*(*x*) = *m*(*x* − 1100) + *n*), 2880 sets of *m* and *n* values were obtained. To obtain multiple sets of dark noise drift data, the reference value for calculating dark noise drift was altered, and the dark noise at the lowest and highest ambient temperatures was utilized as the reference values. These data were processed using the same method to obtain multiple sets of *m* and *n* values.

**Figure 6:**
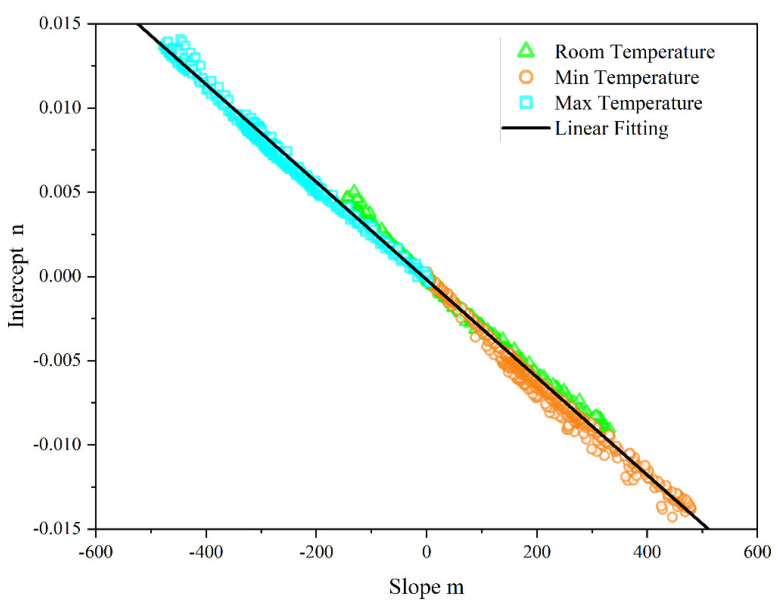
The relation between slope *m* and intercept *n*.

A two-dimensional coordinate system was employed to visualize the relationship between the slope m and intercept n values, and those data were plotted in Figure 6. As can be seen from the figure, the slope *m* decreases as the intercept *n* increases. A linear fitting method was used to analyze the data in the graph, resulting in a fitting equation of slope *m* with respect to intercept *n*.

(8) m=−0.00001×(18.61−2.899n)

The intercept *n* holds significant physical meaning as it represents the dark noise drift value of the fiber optic spectrometer at 1100 nm. Based on Formula (8) and the drift value of dark noise captured by a fiber optic spectrometer at 1100 nm, it is possible to calculate the drift value of dark output noise in the wavelength range of 1200 to 2500 nm. The linearly fitted dark output noise drift signal is employed to correct the silicon carbide radiation signal and determine its spectral emissivity by Formula (5).

**Figure 7:**
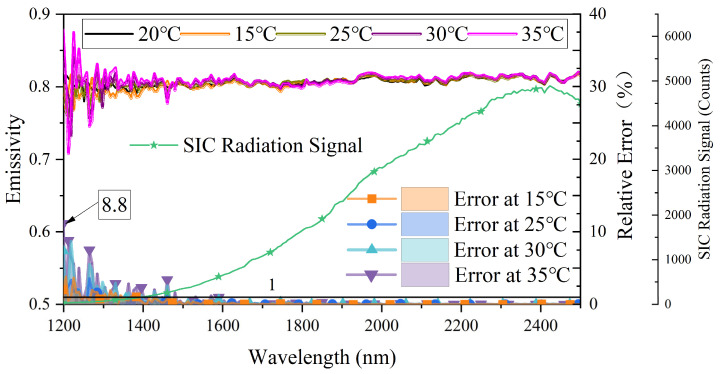
The spectral emissivity of silicon carbide corrected by linear fitting correction scheme at different ambient temperatures and relative error of it.

The silicon carbide emissivity corrected by linear fitting and the relative error of emissivity is illustrated in the upper and lower parts of Figure 7, respectively. Table 2 shows the relative error of corrected emissivity by linear fitting correction scheme at different wavelength ranges. It can be seen from Figure 7 that the corrected emissivity relative error shows a decreasing trend as the wavelength increases. The maximum relative error of corrected emissivity is 8.8%, and all the relative error of corrected emissivity is less than 1% when the wavelength exceeds 1600 nm. The radiation signal of SIC is also plotted in Figure 7. The relative error of silicon carbide emissivity in short wavelength after correction is large, mainly because the signal in this band is too small. It can be seen from Table 2 that as the temperature drift increases, the relative error of the corrected SiC emissivity increases gradually. Table 2 also shows that the relative error of corrected emissivity is less than 0.62% when the wavelength exceeds 1600 nm, and the maximum relative error of corrected emissivity in the wavelength range of 1450–1600 nm is 3.37%. A comparison of Table 1 with Table 2 reveals that the silicon carbide emissivity corrected via linear fitting exhibits significant improvement in contrast to the silicon carbide emissivity corrected via mean value. The maximum relative error of corrected emissivity has been reduced to below 8.8%. In addition, the results of the linear calibration scheme are better than the results of the mean calibration scheme in each band range.

**Figure 8:**
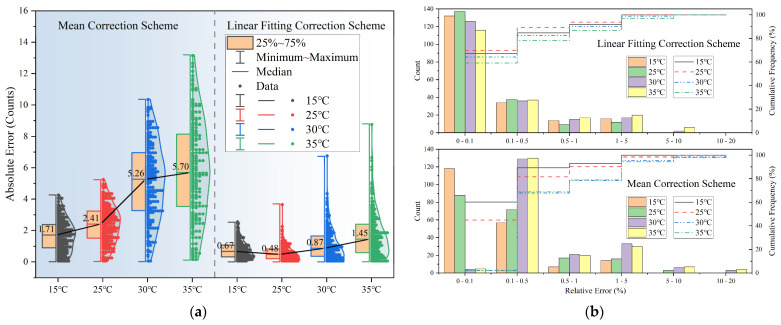
(**a**) The statistical graphics of the absolute error of the radiation signal after the two correction schemes. (**b**) The frequency statistics and cumulative frequency of the corrected silicon carbide emissivity relative errors for the two schemes.

To facilitate a comparison between the results of the linear fitting correction scheme and the mean correction scheme, Figure 8a shows the statistical graphics of the absolute error of the radiation signal after the two correction schemes. The information contained in the figure includes the distribution of the error of the corrected radiation signal, as well as the median value and box plot of the error. It can be seen from the figure that the largest corrected error is approximately 14. However, small sample radiation in the short wavelength causes significant deviation in emissivity. It can be seen from Figure 8a that the error of the linear correction scheme is smaller, and the overall error distribution is concentrated in a range close to 0, while the error of the mean correction scheme is concentrated near the median. Figure 8b displays the frequency statistics and cumulative frequency of the corrected silicon carbide emissivity relative errors for the two schemes. The results show that over half of the silicon carbide emissivity processed using the linear fitting correction scheme has a relative error of less than 0.1%, and nearly 90% of the measured data exhibit a relative error of less than 1%. When the ambient temperature changes little, in other words, when the dark output noise drifts little, the relative error of emissivity utilizing the linear fitting correction scheme does not considerably differ from the relative error of emissivity using the mean correction scheme, and the linear fitting correction scheme slightly outperforms the mean correction scheme. When the ambient temperature varies significantly, the linear fitting correction scheme effectively mitigates the measurement error. Therefore, utilizing the linear fitting correction scheme can substantially reduce measurement errors and enhance measurement accuracy, even for significant ambient temperature changes.

## 6. Conclusions

To improve the accuracy and reliability of emissivity, it is crucial to investigate and address the impact of the dark output noise drift on emissivity. This study experimentally verified that changes in ambient temperature are the primary factor causing dark output noise drift. Building on an improved fiber optic spectrometer, two methods were proposed for characterizing and correcting the dark output noise offset in fiber optic spectrometers: the mean correction scheme and the linear fitting correction scheme. Compared to the mean correction scheme, the linear fitting correction scheme is more effective in solving the issues of dark output noise drift. According to Planck’s radiation law, when the object temperature is low, the spectral radiation is relatively small in the short wavelength. Therefore, even small dark output noise offsets can cause significant measurement errors in emissivity and radiation in the short wavelength. The experimental results indicate that the emissivity of silicon carbide at 550 °C after the mean correction has a relative error of less than 17.2% within the wavelength range of 1200–1450 nm, while the relative error of silicon carbide emissivity after linear fitting correction is less than 8.8%. When the wavelength is greater than 1600 nm, the calibration error of silicon carbide emissivity is below 1% with both correction schemes, among which the mean correction scheme resulted in a calibration error of emissivity lower than 0.8%, while the linear fitting correction scheme resulted in a calibration error of emissivity lower than 0.62%. The corrected relative error for SiC increases as the temperature difference from the ambient temperature at which the dark output noise is first acquired increases. In addition, the radiation error corrected by the linear correction scheme is more concentrated, and the absolute error value is smaller than the mean correction scheme. Overall, this study solved the problem of dark output noise offset in fiber optic spectrometer-based online measurement, providing valuable insights for the development of more accurate and reliable quantitative radiation measurement under complex environments.

## Figures and Tables

**Table 1:** The relative error of corrected emissivity by mean correction scheme.

Ambient Temperature	Relative Error (%)		
	1200–1450 nm	1450–1600 nm	1601–2500 nm
15 °C	4.54	1.49	0.28
25 °C	6.02	2.01	0.38
30 °C	15.33	3.97	0.67
35 °C	17.20	5.06	0.80

**Table 2:** The relative error of corrected emissivity by linear fitting correction scheme.

Ambient Temperature	Relative Error (%)		
	1200–1450 nm	1450–1600 nm	1601–2500 nm
15 °C	3.82	0.86	0.24
25 °C	3.63	1.42	0.18
30 °C	8.71	2.58	0.42
35 °C	8.80	3.37	0.62

## Data Availability

The data in the paper can be obtained from the authors.
